# Efficacy, safety, and predictive model of Palbociclib in the treatment of HR-positive and HER2-negative metastatic breast cancer

**DOI:** 10.1186/s12885-023-11764-8

**Published:** 2024-01-02

**Authors:** Wei Wang, Wenqian Lei, Ziru Fang, Ruiyuan Jiang, Xiaojia Wang

**Affiliations:** 1 Department of Medical Oncology (Breast Cancer), Zhejiang Cancer Hospital, Hangzhou Institute of Medicine (HIM), Chinese Academy of Sciences, 1 Banshandong Road, Gongshu District, Hangzhou, 310022 Zhejiang China; 2 Postgraduate Training Base Alliance of Wenzhou Medical University(Zhejiang Cancer Hospital), Wenzhou Medical University, 270 Xueyuanxi Road, Lucheng District, Wenzhou, 325027 Zhejiang China; 3 Department of Clinical Medicine, Medical College of Nanchang University, 461 Bayi Avenue, Donghu District, Nanchang, 330006 Jiangxi China

**Keywords:** HR + /HER2- breast cancer, Palbociclib, Predictive model, Nomogram, Prognosis

## Abstract

**Purpose:**

This research designeded to: 1. Analyze the efficacy and safety of Palbociclib treatment in HR-positive and HER2-negative (HR + /HER2-) metastatic breast cancer(MBC) patients. 2. Establish and validate a nomogram model for predicting the progression-free survival (PFS) rates of 6 months, 12 months, and 18 months in HR + /HER2- MBC patients after receiving Palbociclib plus endocrine therapy (ET).

**Patients and methods:**

1. This research retrospectively analyzed the efficacy and safety of Palbociclib combined with ET in 214 patients with HR + /HER2- MBC. 2. A nomogram was designed and constructed with the retrospective clinical data of 214 patients with HR + /HER2- MBC who received Palbociclib plus ET at Zhejiang Cancer Hospital in China from August 2018 to August 2022. Among these patients, 161 were randomly assigned to the training cohort, while 53 to the validation cohort. The predictive accuracy of the nomogram was assessed through the analysis the area under the receiver operating characteristic(ROC) curve, calibration curve, and decision curve analysis(DCA).

**Results:**

1. Median PFS was 7.17 months (95% CI: 7.61—10.05 months), with an objective response rate (ORR) of 2.80% and a disease control rate (DCR) of 34.58%. The most prevalent grade 3–4 adverse event was neutropenia (38.79%). 2. Multiple variable analysis of the training set revealed that age < 60 years old, PR < 20%, Ki-67 ≥ 20%, luminal B molecular subtype, primary resistance to ET, receipt of late-stage chemotherapy, and presence of liver metastasis or ≥ 2 visceral metastases were independent prognostic factors associated with poor PFS (*P* < 0.05). Then, the predictive model underwent development and validation utilizing the aforementioned parameters. On the one hand, the area under the ROC curve (AUC) values of the training set at 6 months, 12 months, and 18 months were 0.771, 0.783, and 0.790, respectively, indicating a strong predictive ability of the developed model. On the other hand, the AUC of the validation set at 6 months, 12 months, and 18 months were 0.720, 0.766, and 0.754, respectively, suggesting the favorable discriminatory ability of the model. The calibration curves also exhibited a good fit with the ideal curves, and the DCA demonstrated the clinical applicability of the model. The nomogram's different scores could distinguish PFS.

**Conclusion:**

This retrospective study demonstrates the efficacy of Palbociclib in Chinese breast cancer patients. Moreover, the clinical parameters showed a significant association with the prognosis of HR + /HER2- MBC, and the prognostic models constructed based on these variables also displayed robust predictive power, which could offer more intuitive and convenient references for clinical doctors to formulate follow-up treatment plans.

**Supplementary Information:**

The online version contains supplementary material available at 10.1186/s12885-023-11764-8.

## Introduction

According to statistics, hormone receptor-positive, human epidermal growth factor receptor 2 negative (HR + /HER2-) breast cancer comprises around 76% of all breast cancer cases [1]. Loss of control over the cell cycle is one of the primary causes of cancer, and this is also a critical target for drug therapy [2]. As is well documented, cyclin-dependent kinases 4/6 (CDK4/6) phosphorylate the retinoblastoma protein (Rb) by binding to cyclin D, thereby releasing transcription factor E2F and allowing the smooth progression of the cell cycle from the G1 phase to the S phase [3]. So far, combining a CDK4/6 inhibitor (CDK4/6i) with endocrine therapy (ET) has emerged as the established treatment approach for this form of breast cancer [4, 5]. Regrettably, despite the significant advancements in survival observed with the use of CDK4/6i in combination with ET in recent years, HR + /HER2- metastatic breast cancer (MBC) remains an incurable disease. As the first CDK4/6i to be marketed in China, Palbociclib has been extensively used in the clinical setting. Furthermore, multiple trials have showcased the efficacy and safety of CDK4/6i in treating breast cancer. Indeed, its licensure has brought hope to HR + /HER2- breast cancer patients, and its therapeutic effect is widely recognized. Meanwhile, effective prediction methods are also necessary [6–9]. Some factors, such as molecular subtypes, sites of metastasis, and prior treatment, could impact the survival of MBC patients and may assist in formulating an individualized therapeutic treatment strategy. Nonetheless, there is a scarcity of predictive models to specifically designed to assess potential prognostic factors for HR + /HER2- MBC [10, 11].

Therefore, developing a predictive model that can integrate multiple variables is a pivotal step in providing drug references for Chinese HR + /HER2- MBC patients. This research designeded to retrospectively analyze the data of 214 patients with HR + /HER2- MBC who received Palbociclib plus ET at Zhejiang Cancer Hospital and explore the efficacy and safety of these drugs in this population. By analyzing the clinical parameters, researchers developed and validated a predictive nomogram model capable of estimating the effectiveness of the treatment regimen in HR + /HER2- MBC patients.

## Patients and methods

### Study design and population

The research retrospectively examined data pertaining to patients with HR + /HER2- MBC who underwent Palbociclib therapy in conjunction with ET treatment at Zhejiang Cancer Hospital between August 2018 and August 2022. The inclusion criteria were as follows (1): individuals who were diagnosed with HR + /HER2- MBC within the time frame of August 2018 to August 2022, encompassing both de novo stage IV disease and relapsed disease, and who had undergone a minimum of one cycle of Palbociclib in combination with ET treatment. (2): complete medical information, including age upon diagnosis, estrogen receptor(ER) or progesterone receptor(PR) status, HER2 status, distant metastatic sites, local therapy, treatment approach, the Palbociclib combined with ET treatment plan, the initial Palbociclib dosage, and adverse reactions encountered during Palbociclib treatment. The exclusion criteria were as follows (1): lack of accessible medical records and presence of coexisting cancers, (2): positive HER2 status (3): both ER and PR status being negative, and (4): diagnosis of triple-negative breast cancer.

**Fig. 1:**
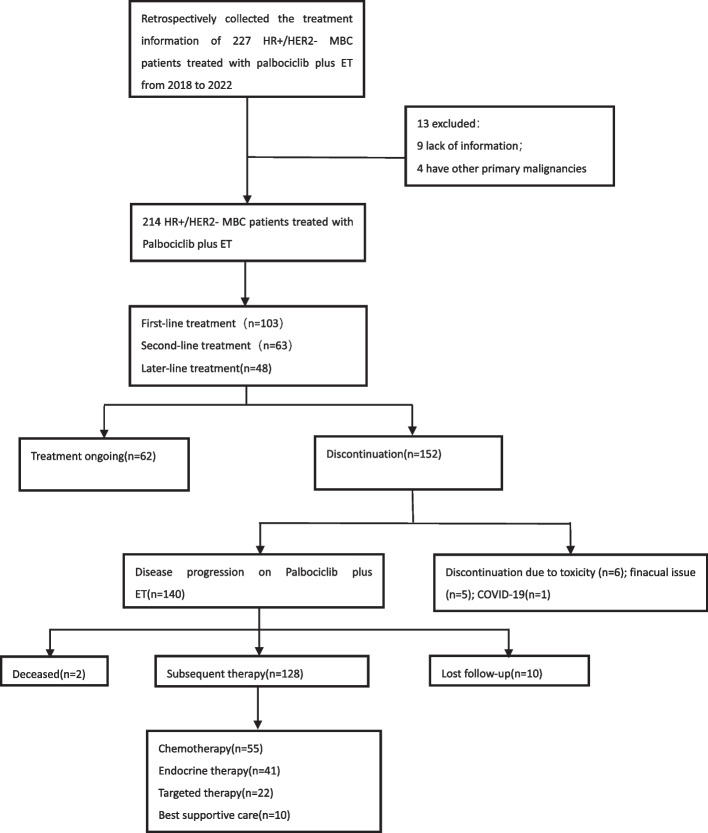
Study flow chart

Two hundred twenty seven patients were eligible to participate in this research. After excluding patients with missing information (*n* = 9) or other primary malignant tumors (*n* = 4), 214 patients were finally included in the analysis. The flowchart of this retrospective study is displayed in Fig. 1. The Medical Ethics Committee of Zhejiang Cancer Hospital granted approval for this research. Informed consent was waived by the Medical Ethics Committee of Zhejiang Cancer Hospital due to the retrospective nature of this study.

### Data collection

Medical record data has been retrieved, comprising age, menstrual status, ER or PR status, HER2 status, history of adjuvant therapy, tumor metastasis organs, and number of metastatic sites, Palbociclib combined with ET treatment plan, past treatment plan, dose adjustment, efficacy, adverse reactions, etc. Progression-free survival (PFS) served as the primary endpoint in the study, while the objective response rate (ORR) and disease control rate (DCR) acted as the secondary endpoints. PFS was defined as the time interval (in months) from the initiation of CDK4/6i to the first recorded clinical and/or radiographic evidence of disease progression or death from any cause (whichever occurs earlier). Disease progression (PD) was defined as the beginning of radiographic progression, clinical progression, or the initiation of a new treatment line for MBC. A new treatment line was defined as a change in the treatment regimen or starting a new chemotherapy, endocrine therapy, or targeted therapy.

According to RECIST 1.1, the therapeutic effect after receiving Palbociclib was classified into four categories: Complete Response (CR), Partial Response (PR), Stable Disease (SD), and Progressive Disease (PD). ORR was defined as the percentage of patients achieving CR or PR, while DCR was defined as the proportion of patients achieving CR, PR, or SD. HR + /HER2- breast cancer cases were categorized into luminal A (PR ≥ 20% and Ki-67 < 20%) and luminal B (PR < 20% and Ki-67 ≥ 20%) [12]. Because guidelines of Chinese Society of Clinical Oncology(CSCO) breast cancer 2022 doesn’t give margin for Ki-67, the authors chose the median as the boundary value. All patients were followed up in October 2022.

### Treatments

Based on the treatment regimens involving Palbociclib, patients were categorized into three subgroups. These subgroups underwent Palbociclib administration in combination with ET, serving as the first-line, second-line, and later-line therapies for MBC. The PFS of each subgroup was separately analyzed to observe differences among each overall population. As the first CDK4/6i to be approved in China, Palbociclib has been widely employed in HR + /HER2- MBC patients. More specifically, it is usually administered in combination with aromatase inhibitors (AIs) or Fulvestrant. The typical initial dose of Palbociclib is 125 or 100 mg/day [13]. CSCO recommends an initial dose of 125 mg/day (Palbociclib is typically taken once daily for a duration of three weeks, followed by a one-week interval) [14]. However, doctors can adjust the dosage and timing of the medication based on the patient's age and physical condition.

### Statistical analysis

Two hundred and fourteen HR + /HER2- MBC patients were randomly allocated in a 3:1 ratio to either the training set (*n* = 161) or validation set (*n* = 53) by using R version 4.1.2 (http://www.r-project.org/). Then, the independent samples t-test or chi-square test was used to evaluate differences in variables between the training and validation sets. In the training set, univariate and multivariate analyses were conducted using SPSS version 25.0 to determine variables significantly associated with PFS and to calculate the hazard ratio (HR) and 95% confidence interval(CI) for each variable. Utilizing the findings from a comprehensive multivariate analysis, a predictive nomogram model was subsequently established. The discriminative ability of the established model was assessed using the receiver operating characteristic (ROC) curve and the area under the ROC curve (AUC). Afterward, the performance of the nomogram was evaluated in the validation set by using 200-fold cross-validation in R. Furthermore, R was utilized to generate calibration plots for the training and validation sets, which can visually measure the degree of closeness between the actual conditions and the predicted conditions of the nomogram. At the same time, The programming language R was employed to generate decision curves, thereby assessing the predictive efficacy of the model within the clinical context. A two-tailed *P*-value below 0.05 was deemed to have statistical significance.

## Results

### Patient characteristics and treatment patterns

The research's median follow-up duration spanned 28.6 months (95% CI, 22.0–35.2 months). Among the 214 patients, 62.62% (134/214) were under 60 years old, with a mean age of 57 years. 82.24% (176/214) of HR + /HER2- MBC patients were already postmenopausal prior to the administration of Palbociclib in combination with ET. 37/214 patients (17.29%) were diagnosed as stage IV at their first diagnosis. In the latest immunohistochemistry analysis prior to using Palbociclib, more than half of the patients had PR < 20% (113/214, 52.80%) and Ki-67 ≥ 20% (119/214, 55.61%). Notably, the most common histological type among these patients was invasive ductal carcinoma (163/214, 76.17%), and the most common molecular subtype was luminal B (135/214, 63.08%). 85.51% (183/214) of patients underwent surgical intervention. At the same time, 57.01% (122/214) of the patients received adjuvant radiotherapy, whereas 78.04% (167/214) of the patients received adjuvant endocrine therapy. Furthermore, 42.52% (91/214) of patients received previous systemic chemotherapy before using Palbociclib. Patients were classified into primary resistance and acquired resistance based on whether they received adjuvant endocrine therapy for more than 2 years or first-line endocrine therapy for more than 6 months. Patients who experienced recurrence after 1 year after completion of adjuvant endocrine therapy or who had not received any prior endocrine therapy were considered sensitive to endocrine therapy [15]. In this study, 69.16% (148/214) of patients developed primary resistance to ET, while only 30.84% (66/214) were sensitive to ET or developed secondary resistance. The median PFS of Palbociclib was 7.17 months (95% CI, 7.61 months to 10.05 months). Prior to the use of Palbociclib in combination with ET, 63.55% (136/214) of patients had already developed visceral metastasis, and 55.61% (119/214) had ≥ 2 metastatic sites. Fortunately, only 34.58% (74/214) of patients manifested liver metastasis. Prior studies have reported that patients with liver metastasis who were given Palbociclib in combination with ET have a lower treatment efficacy than those without. Finally, 64.95% (139/214) of patients had a disease-free survival (DFS) period of more than 24 months.

The number of patients who received treatment involving the combination of Palbociclib and ET as first-line, second-line, and later-line therapies were 103/214 (48.13%), 63/214 (29.44%), and 48/214 (22.43%), respectively. Moreover, 97.20% (208/214) of patients received an initial dose of 125 mg/day of Palbociclib, while only 2.80% (6/214) of patients were initiated on a dose of 100 mg/day. Lastly, 42.06% (90/214) of patients had their dosage adjusted owing to intolerable side effects, with the most common side effect leading to dosage adjustment being grade 3/4 neutropenia. The most commonly co-administered ET drug with Palbociclib was AIs (131/214, 61.21%), followed by Fulvestrant (83/214, 38.79%). 152/214 (71.03%) patients discontinued combination treatment during the study period. Among them, 92.11% (140/214) switched to another regimen due to disease progression, 5/214 (3.29%) patients discontinued treatment due to financial reasons, 6/214 (3.95%) due to intolerable adverse reactions, and 1/214 (0.66%) due to the COVID-19 pandemic. Detailed clinical characteristics are presented in Table 1.

**Table 1:** Clinical characteristics in HR + /HER2- MBC

**Clinical characteristics**	**Cases**	**First-line** **(103)**	**Secone-line** **(63)**	**Later-lines** **(48)**	**Statistic**	***P***
PFS, M (Q_1_, Q_3_)	7.17 (7.61–10.05)	12.13 (5.47–19.62)	8.93 (4.22–16.13)	5.47 (2.74–9.43)	χ^2^ = 17.115	< .001
Age(years), n(%)					χ^2^ = 1.324	0.516
< 60	134 (62.62%)	63 (61.17%)	43 (68.25%)	28 (58.33%)		
≥ 60	80 (37.38%)	40 (38.83%)	20 (31.75%)	20 (41.67%)		
Menopausal status					χ^2^ = 2.665	0.264
Premenopausal or Perimenopausal	38 (17.76%)	19 (18.45%)	14 (22.22%)	5 (10.42%)		
Postmenopausal	176 (82.24%)	84 (81.55%)	49 (77.78%)	43 (89.58%)		
IV stage at first diagnosis						
Yes	37 (17.29%)	23 (22.33%)	8 (12.70%)	6 (12.50%)		
No	177 (82.71%)	80 (77.67%)	55 (87.30%)	42 (87.50%)		
PR					χ^2^ = 0.337	0.845
< 20%	113 (52.80%)	63 (61.17%)	31 (49.21%)	19 (39.58%)		
≥ 20%	101 (47.20%)	40 (38.83%)	32 (50.79%)	29 (60.42%)		
Ki-67					χ^2^ = 1.181	0.554
< 20%	95 (44.39%)	45 (43.69%)	31 (49.21%)	19 (39.58%)		
≥ 20%	119 (55.61%)	58 (56.31%)	32 (50.79%)	29 (60.42%)		
Histologic type of tumor					χ^2^ = 3.267	0.195
Invasive ductal carcinoma	163 (76.17%)	72 (69.90%)	51 (80.95%)	40 (83.33%)		
Invasive lobular carcinoma	51 (23.83%)	31 (30.10%)	12 (19.05%)	8 (16.67%)		
Molecular type of tumor					χ^2^ = 0.472	0.79
Luminal A	79 (36.92%)	42 (40.78%)	21 (33.33%)	16 (33.33%)		
Luminal B	135 (63.08%)	61 (59.22%)	42 (66.67%)	32 (66.67%)		
Prior surgeries						
Yes	183 (85.51%)	82 (79.61%)	57 (90.48%)	44 (91.67%)		
No	31 (14.49%)	21 (20.39%)	6 (9.52%)	4 (8.33%)		
Adjuvant radiotherapy					χ^2^ = 8.339	0.015
Yes	122 (57.01%)	65 (63.11%)	45 (71.43%)	12 (25.00%)		
No	92 (42.99%)	38 (36.89%)	18 (28.57%)	36 (75.00%)		
Adjuvant endocrine therapy					χ^2^ = 25.831	< 0.01
Yes	167 (78.04%)	65 (63.11%)	58 (92.06%)	44 (91.67%)		
No	47 (22.96%)	38 (36.89%)	5 (7.94%)	4 (8.33%)		
Prior chemotherapy for MBC					χ^2^ = 65.661	< 0.01
Yes	91 (42.52%)	16 (15.53%)	36 (57.14%)	39 (81.25%)		
No	123 (57.48%)	87 (84.47%)	27 (42.86%)	9 (18.75%)		
Sensitivity to endocrine therapy					χ^2^ = 0.225	0.894
Sensitivity or acquired resistance	66 (30.84%)	61 (59.22%)	4 (6.35%)	1 (2.08%)		
Primary resistance	148 (69.16%)	42 (40.78%)	59 (93.65%)	47 (97.92%)		
Visceral metastasis					χ^2^ = 2.651	0.266
Yes	136 (63.55%)	53 (51.46%)	47 (74.60%)	36 (75.00%)		
No	78 (36.d45%)	50 (48.54%)	16 (25.40%)	12 (25.00%)		
Metastatic sites						
Bone only	30 (14.02%)	16 (15.53%)	11 (17.46%)	3 (6.25%)	χ^2^ = 0.432	0.806
Lung involvement	86 (40.19%)	33 (32.04%)	30 (47.62%)	23 (47.92%)	χ^2^ = 4.811	0.09
Lymph node involvement	110 (51.40%)	54 (52.43%)	25 (39.68%)	31 (64.58%)		
Brain involvement	10 (4.67%)	2 (1.94%)	4 (6.35%)	4 (8.33%)	-	0.141
Number of sites for visceral metastasis					χ^2^ = 13.121	0.001
< 2	95 (44.39%)	56 (54.37%)	28 (44.44%)	11 (22.92%)		
≥ 2	119 (55.61%)	47 (45.64%)	35 (55.56%)	37 (77.08%)		
Hepatic metastases					χ^2^ = 1.336	0.505
Yes	74 (34.58%)	27 (26.21%)	23 (36.51%)	24 (50.00%)		
No	140 (65.42%)	76 (73.79%)	40 (63.49%)	24 (50.00%)		
Disease-free survival						
< 24 months	38 (17.76%%)	18 (17.48%)	11 (17.46%)	9 (18.75%)		
≥ 24 months	139 (64.95%)	62 (60.19%)	44 (69.84%)	33 (68.75%)		
De-novo stage IV	37 (17.29%)	23 (22.33%)	8 (12.70%)	6 (12.50%)		
Initial dose of Palbociclib						
125 mg/d	208 (97.20%)	103 (100%)	59 (93.65%)	47 (97.92%)		
100 mg/d	6 (2.80%)	0 (0.0)	4 (6.35%)	1 (2.08%)		
Combined endocrine therapy					χ^2^ = 0.482	0.786
Aromatase inhibitors	131 (61.21%)	73 (70.87%)	34 (53.97%)	24 (50.00%)		
Fulvestrant	83 (38.79%)	30 (29.13%)	29 (46.03%)	24 (50.00%)		
Treatment discontinuation						
Yes	152 (71.03%)	59 (57.28%)	49 (77.78%)	44 (91.67%)		
No	62 (28.97%)	44 (42.72%)	14 (22.22%)	4 (8.33%)		
Reasons for treatment discontinuation						
Disease progression	140 (92.11%)	53 (89.83%)	45 (91.84%)	42 (95.45%)		
Financial issue	5 (3.29%)	2 (3.39%)	2 (4.08%)	1 (2.27%)		
Toxicity	6 (3.95%)	3 (5.08%)	2 (4.08%)	1 (2.27%)		
COVID-19	1 (0.66%)	1 (1.69%)	0 (0.0)	0 (0.0)		

### Efficacy of Palbociclib plus ET

During a median follow-up duration of 28.6 months (95% CI, 22.0–35.2 months), 140 patients experienced disease progression. The median PFS of patients on Palbociclib combined with ET was 7.17 months (95% CI, 7.61–10.05 months). All lesions were measurable, with no CR cases, 6 PR cases, 68 cases of SD, and 140 cases of PD. The DCR was 34.58%, and the ORR was 2.80%.

According to the RECIST1.1 criteria for tumor assessment, all lesions in this retrospective study were measurable. Among all patients, 2.80% (6/214) had a tumor assessment of PR, 31.78% (68/214) achieved SD, and 65.42% (140/214) achieved PD. Among them, the ORR and DCR of HR + /HER2- MBC patients using Palbociclib in combination with ET as first-line treatment were 5.83% and 48.54%, respectively. Interestingly, the ORR and DCR of second-line and later-line treatments were 0.0% and 28.57%, and 0.0% and 12.50%, respectively. Detailed tumor evaluations for the different treatment groups are listed in Table 2.

**Table 2:** Treatment response

Best treatment evaluation n (%)	Cases	First-line	Second-line	Later-lines
CR	0 (0.0)	0 (0.0)	0 (0.0)	0 (0.0)
PR	6 (2.80%)	6 (5.83%)	0 (0.0)	0 (0.0)
SD	68 (31.78%)	44 (42.72%)	18 (28.57%)	6 (12.50%)
PD	140 (65.42%)	53 (51.46%)	45 (71.43%)	42 (87.50%)
ORR	2.80%	5.83%	0.0	0.0
DCR	34.58%	48.54%	28.57%	12.50%

*Abbreviations*: *CR* Complete response, *PR* Partial response, *SD* Stable disease, *PD* Progressive disease, *ORR* Objective response rate, *DCR* Disease control rate

The most prevalent grades 3–4 adverse reaction was neutropenia (38.79%), which had a lower incidence compared with that of the Paloma series studies but comparable with previous studies (Table 3). After using Palbociclib combined with ET for a period of time, 65.42% (140/214) of HR + /HER2- MBC patients experienced disease progression. Herein, electronic medical records were reviewed, and telephone follow-ups were conducted to gain a better understanding of their treatment regime following disease progression. After excluding lost-to-follow-up and death cases, 42.97% (55/128) were observed to choose chemotherapy as the next treatment modality, with Abraxane being the most commonly selected chemotherapeutic drug. Additionally, 32.03% (41/128) chose to continue with endocrine therapy, while 17.19% (22/128) chose targeted therapy. The patients' survival time was also extended to varying degrees (Fig. 1).

**Table 3:** Grade 3–4 adverse events

Grade 3–4 adverse events	Case (n)	%
Neutropenia	83	38.79
Leukopenia	62	28.97
Anemia	5	2.34
Thrombocytopenia	15	7.01
Diarrhea	1	0.47

### Influencing factors associated with PFS in the training cohort

After conducting a univariate analysis on the training dataset, the following variables were noted to be statistically significant (*P* < 0.05): age, PR, Ki-67, molecular subtype, adjuvant radiotherapy or endocrine therapy, late-stage chemotherapy, sensitivity to endocrine therapy, number of visceral metastases, presence of liver metastases, and number of Palbociclib treatment lines. After incorporating the aforementioned parameters into a multivariate Cox proportional hazards regression model, patients aged < 60 years old, PR < 20%, Ki-67 ≥ 20%, luminal B molecular subtype, primary resistance to ET, receipt of late-stage chemotherapy, and presence of liver metastasis or ≥ 2 visceral metastases were identified as independent prognostic factors associated with poor PFS (*P* < 0.05) (Table 4, Supplementary Fig. 1).

**Table 4:** Univariate and multivariate analyses of PFS

**Clinical characteristics**	**Univariate analysis**	**Multivariate analysis**	
	***P* value**	**HR (95% CI)**	***P* value**
Age	< 0.01	0.483 (0.320–0.731)	< 0.01*
Menopausal status	0.533		
PR	< 0.01	0.595 (0.404–0.875)	< 0.01*
Ki-67	< 0.01	1.895 (1.266–2.836)	< 0.01*
Pathological typing at initial diagnosis	0.376		
Molecular typing at initial diagnosis	< 0.01	1.563 (1.044–2.340)	0.029*
Adjuvant radiotherapy	< 0.01		0.660
Prior chemotherapy for MBC	< 0.01	2.104 (1.438–3.078)	< 0.01*
Adjuvant endocrine therapy	0.049		0.825
Sensitivity to endocrine therapy	< 0.01	0.458 (0.284–0.738)	< 0.01*
Visceral metastasis	0.251		
Number of sites for visceral metastasis	0.044	1.559 (1.059–2.295)	0.023*
Hepatic metastases	< 0.01	2.172 (1.467–3.217)	< 0.01*
Combined endocrine therapy	0.440		
Line of Palbociclib plus ET	< 0.01		0.076

*Abbreviations*: *MBC* metastatic breast cancer, *ET* endocrine therapy

^*^p < 0.05

### Nomogram construction and validation

**Fig. 2:**
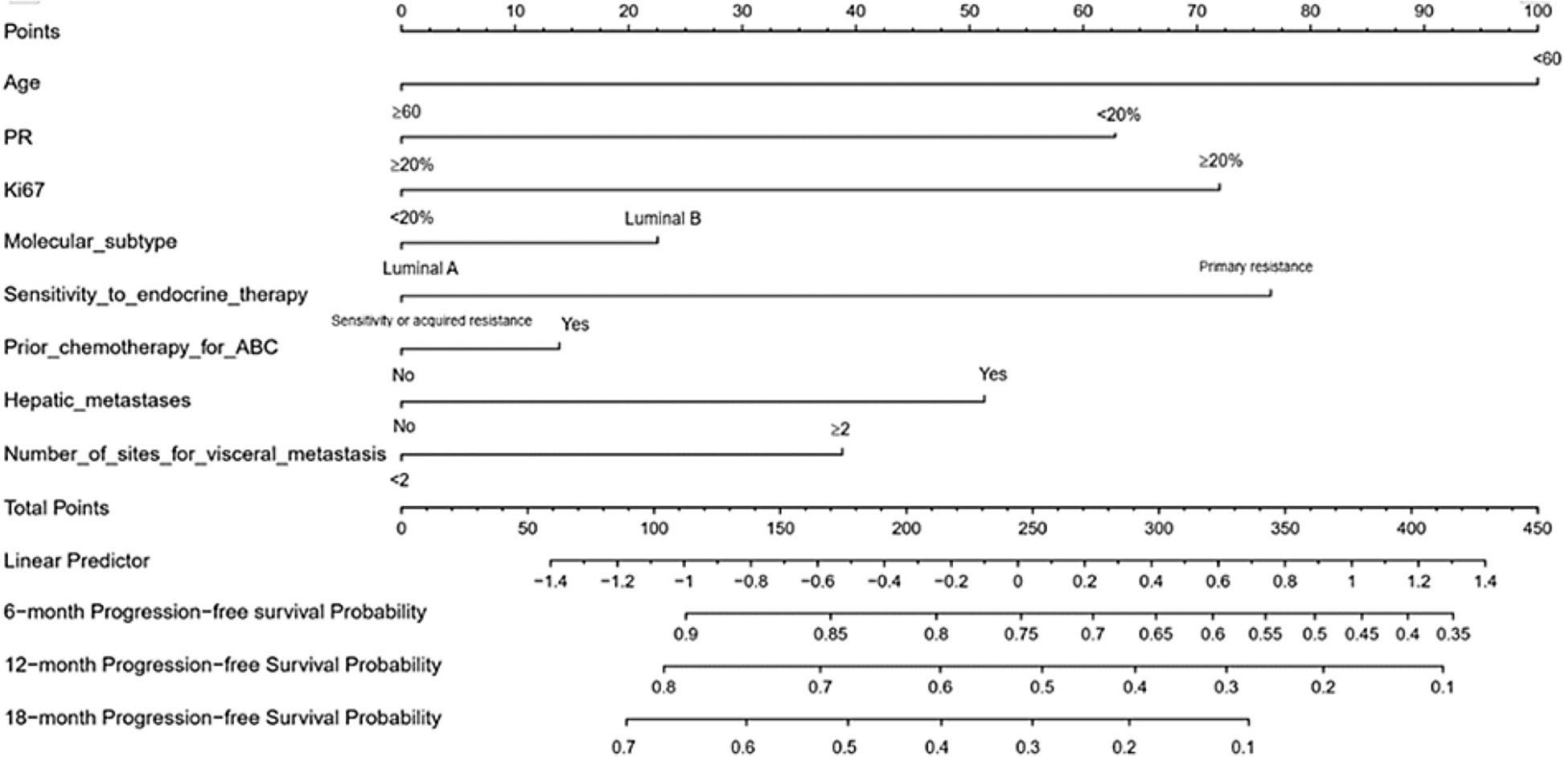
Nomogram for predicting PFS after Palbociclib plus ET treatment. To calculate the probability of PFS, each predictor is assigned a score on the points axis corresponding to its location on the axis. The scores of each predictor are summed up on the total points axis, which represents the probability of 6-, 12-, or 18-month PFS

**Fig. 3:**
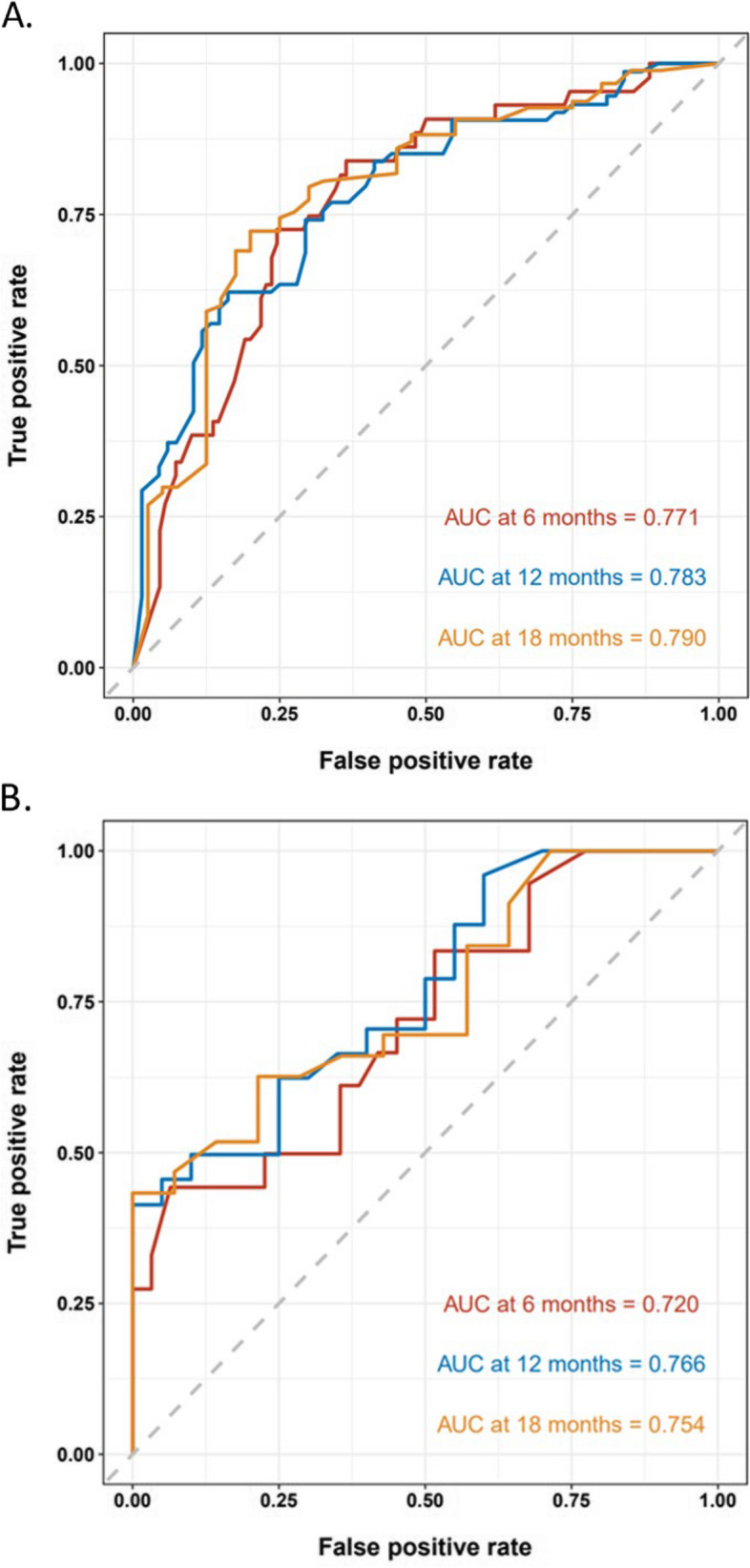
The discrimination of the nomogram was assessed using ROC curves in both the training cohort (**A**) and the validation cohort (**B**)

**Fig. 4:**
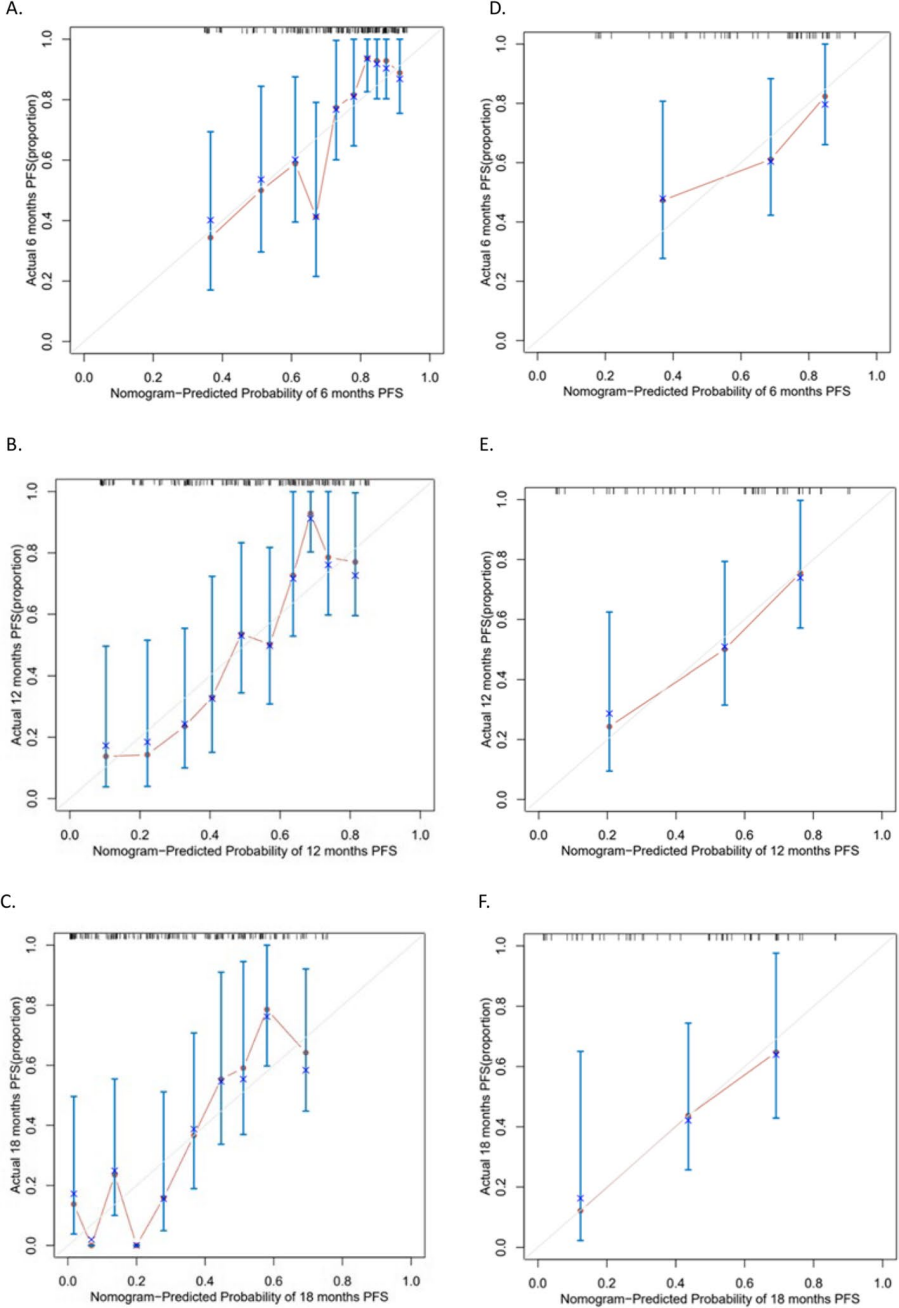
The calibration plot curve for PFS prediction in the training cohort at (**A**) 6 months, (**B**) 12 months and (**C**) 18 months and in the validation cohort at (**D**) 6 months, (**E**) 12 months and (**F**) 18 months. The x-axis signifies the predicted probability of PFS from the nomogram, while the y-axis exhibits the corresponding actual survival probability

**Fig. 5:**
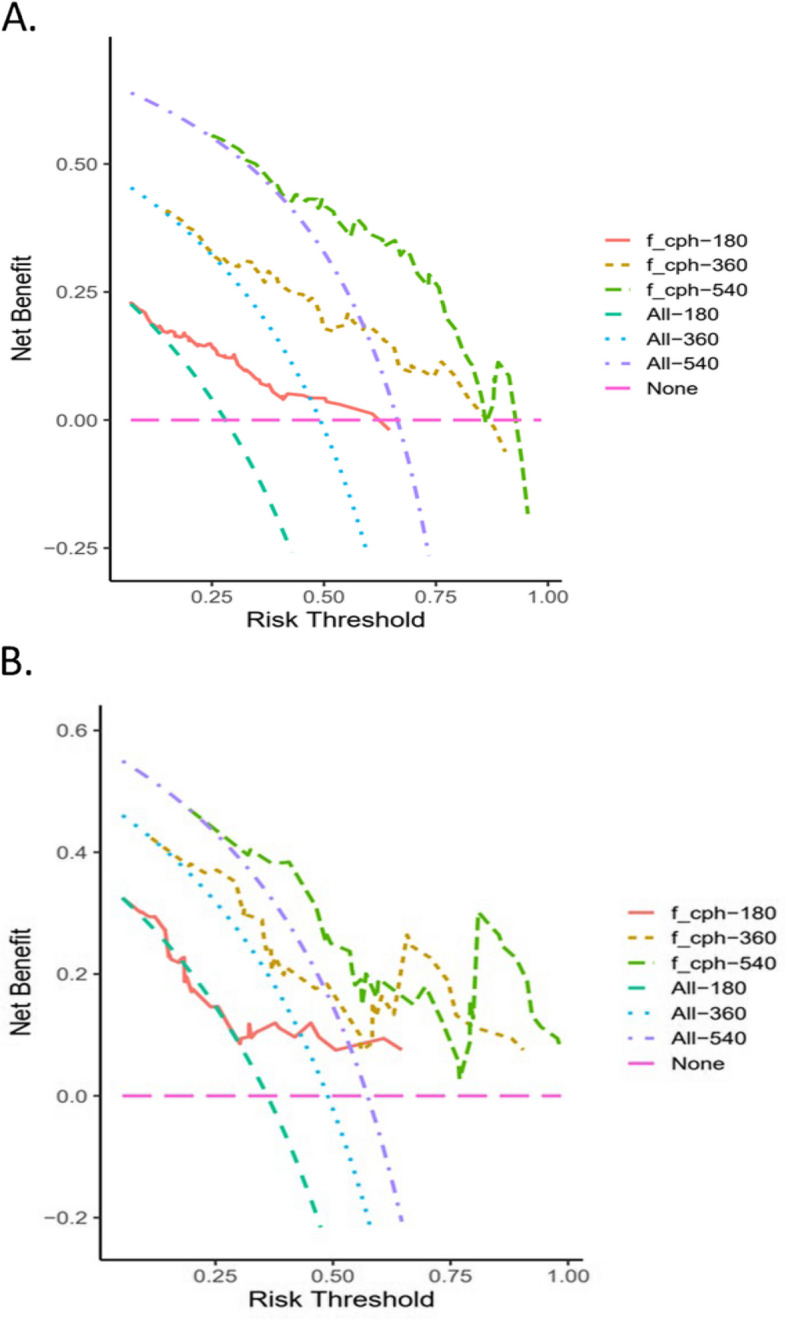
Clinical benefits in the (A)training cohort and (B)validation cohort. The decision curve analysis highlights the practical value of the nomogram for PFS. On the x-axis, there is a range of risk potential thresholds probabilities for PFS, while the y-axis represents the net benefit derived from the model. The orange line, brown and green dotted curve represents the predictive nomogram for 6-, 12-, 18-month PFS, respectively. The cyan,blue and purple dotted curve assumes intervention for all and the pink dotted curve represents intervention for none

A nomogram model was established based on the independent prognostic factors identified from multivariate analysis. As displayed in Fig. 2, the specific scores for the eight variables were summed to obtain a total score, which was then used to determine the corresponding PFS rates of 6 months, 12 months, and 18 months. In addition, the study examined the discriminative performance of the nomogram model via AUC. On the one hand, the AUC values for PFS at 6, 12, and 18 months were 0.771, 0.783, and 0.790 in the training set, respectively (Fig. 3A), indicating the strong predictive ability of the model. On the other hand, the AUC values for PFS at 6, 12, and 18 months were 0.720, 0.766, and 0.754 in the validation set, respectively (Fig. 3B), implying favorable discriminative ability. The calibration curve also delineated a goodness-of-fit with the ideal curve (Figs. 4A-F). Moreover, decision curve analysis (DCA) was employed to compare the clinical effectiveness of various predictive models. The x-axis illustrates a range of probability thresholds for PFS representing varying levels of risk potential, while the y-axis denotes the net benefit derived from the model. The DCA denoted that the model brought clinical benefits to patients. In the training set, the DCA at 6, 12, and 18 months depicted clinical benefit within the risk threshold of 0.15–0.65, 0.30–0.90, and 0.55–0.90, respectively. In the validation set, the DCA at 6, 12, and 18 months showed clinical benefit within the risk threshold of 0.30–0.65, 0.25–0.85, and 0.30–0.95, respectively (Figs. 5A-B).

## Discussion

The onset and progression of breast cancer are influenced by multiple factors. The entire pathogenic process is intricate and variable, with each variable playing a greater or lesser role. Although studies have demonstrated that hematological parameters and genetic factors can predict the prognosis of HR + /HER2- MBC patients, these studies are limited to a single biomarker, and their predictive ability is limited [16–18]. In the era of precision medicine, the promising direction lies in the combination of multiple variables and even interdisciplinary cooperation. In the present study, the predictive nomogram model was built on the basis of multivariate analysis, which combined all statistically significant variables so as to accurately predict the prognosis of HR + /HER2- MBC individuals following treatment with Palbociclib in combination with ET. The nomogram, similar to the Tumor, Node, Metastasis (TNM) staging system, can provide clinicians with a more intuitive way to assess patients' prognosis and provide a basis for subsequent medication use.

This retrospective study collected data from 214 HR + /HER2- MBC patients at the Zhejiang Cancer Hospital. Univariate and multivariate analyses were performed, and aged < 60 years old, PR < 20%, Ki-67 ≥ 20%, luminal B molecular subtype, primary resistance to ET, receipt of late-stage chemotherapy, and presence of liver metastasis or ≥ 2 visceral metastases were determined to be independent prognostic factors associated with shorter PFS. The nomogram's validation showcased exceptional predictive capacity in regards to differentiation and calibration. Besides, Uniformity was noted when contrasting our model with other previously published models, signifying the significance of our nomogram in clinical practice. In other studies [19–22], adjuvant radiotherapy, visceral metastasis, and ET lines were also identified as independent prognostic factors, although these variables did not influence PFS in this study. Disparities in the findings between our study and prior research indicate the heterogeneity of MBC. Hence, HER2-positive breast cancer and triple-negative breast cancer patients were excluded from this study to unveil more precise and targeted prognostic factors for the HR + /HER2- MBC subtype. In this study, eight variables were inputted for the development of the nomogram: age, PR, Ki-67, molecular subtype, resistance to ET, late-stage chemotherapy, and liver metastasis or the number of visceral metastases. Age is an independent prognostic factor, as well as the most significant predictor of PFS in HR + /HER2- MBC; patients younger than 60 years old have shorter PFS, which is consistent with previous research results, thereby supporting the hypothesis that breast cancer in younger women exhibits more aggressive behavior, even within HR + /HER2- tumors. Future research should explore the causes of poorer survival in order to develop strategies to improve outcomes in the younger age group [23]. Our study identified Ki-67 as a very significant predictor of PFS, which was in line with the findings of Lee et al. [24]. Indeed, functioning as a proliferation index, Ki-67 has gained widespread recognition and approval for distinguishing luminal A and luminal B breast cancer.

Discordance in HR and HER2 expression levels between primary and recurrent diseases have been reported rates ranging from 3.4% to 60% [25, 26]. So when enrolling patients in this study, the latest immunohistochemical results were used to ensure reliability. When it comes to treating patients with HR + breast cancer, opting for endocrine therapy is a sensible decision. However, HER2- is typically classified as either HER2 non-expressing or HER2 low-expressing, and fluorescence in situ hybridization (FISH) test results are frequently negative. Therefore, the level of HER2 expression may also have an impact on the outcome of individuals with HR + breast cancer. Prior studies [27–29] have also evinced that HER2 non-expression or low-expression has a specific influence on the survival of HR + breast cancer, although not statistically significant. This phenomenon deserves further investigation in the future and may be incorporated into predictive models to more accurately assess the survival prognosis of HR + /HER2- MBC. Nevertheless, based on the data collected from real-world medical practices, a significant number of patients with HR + /HER2- MBC opted for chemotherapy as their first-line and second-line systemic treatment. The factors influencing treatment options are multifaceted. Furthermore, MBC patients also accept other locoregional treatment, including surgical intervention, radiotherapy, radiofrequency ablation, and interventional therapy, which are supplements to systemic treatment. Appropriate local treatment has the potential to relieve patients' pain, effectively manage potentially life-threatening complications, and provide patients with the chance to undergo additional rounds of systemic therapy. However, there is currently no agreement on the optimal timing and criteria for administering locoregional treatment. This requires the input of a multidisciplinary team.

This study also has limitations that need to be taken into account. To begin, this study was limited by its retrospective, single-center design. During the process of collecting medical records, selection bias may have occurred, which could have led to differences in the multivariate analysis. Secondly, duing to the single-center nature of the study, it was not possible to externally validate the nomogram. Lastly, evidence from previous prospective studies was limited and predictive variables were lacking. Therefore, we intend to carry out a more comprehensive prospective analysis using genomics and radiomics in conjunction with proteomics so as to more accurately predict the prognosis of HR + /HER2- MBC patients. Finally, more non-cancer parameters can be analyzed to compare pre-cancerous lesions and breast cancer, with the aim of providing a reference for the timely diagnosis of breast cancer.

## Conclusion

In short, in the context of precision medicine, prognostic models developed based on clinical parameters can provide more intuitive and convenient references for clinical doctors to formulate follow-up treatment plans.

### Supplementary Information

**Additional file 1: Supplementary Fig. 1.** PFS comparisons in subgroups.

## Data Availability

All data generated or analyzed during this study are included in this published article. The data that support the findings of this study are available from the corresponding author, Xiaojia Wang, upon reasonable request.

## Abbreviations

HR + /HER2-: Hormone receptor-positive, human epidermal growth factor receptor 2 negative
MBC: Metastatic breast cancer
PFS: Progression-free survival
ET: Endocrine therapy
ROC: Receiver operating characteristic
DCA: Decision curve analysis
ORR: Objective response rate
DCR: Disease control rate
AUC: Area under the ROC curve
CDK4/6: Cyclin-dependent kinases 4/6
Rb: Retinoblastoma protein
CDK4/6i: CDK4/6 inhibitor
ER: Estrogen receptor
PR: Progesterone receptor
PD: Disease progression
CR: Complete Response
PR: Partial Response
SD: Stable Disease
AIs: Aromatase inhibitors
CSCO: Guidelines of Chinese Society of Clinical Oncology
HR: Hazard ratio
CI: Confidence interval
TNM: Tumor, Node, Metastasis
FISH: Fluorescence in situ hybridization
