# Supplementary figures and images for: Efficacy, safety, and predictive model of Palbociclib in the treatment of HR-positive and HER2-negative metastatic breast cancer
Source: BMC Cancer. 2024 Jan 2;24:1. doi: 10.1186/s12885-023-11764-8 (PMC10762987; doi:10.1186/s12885-023-11764-8)

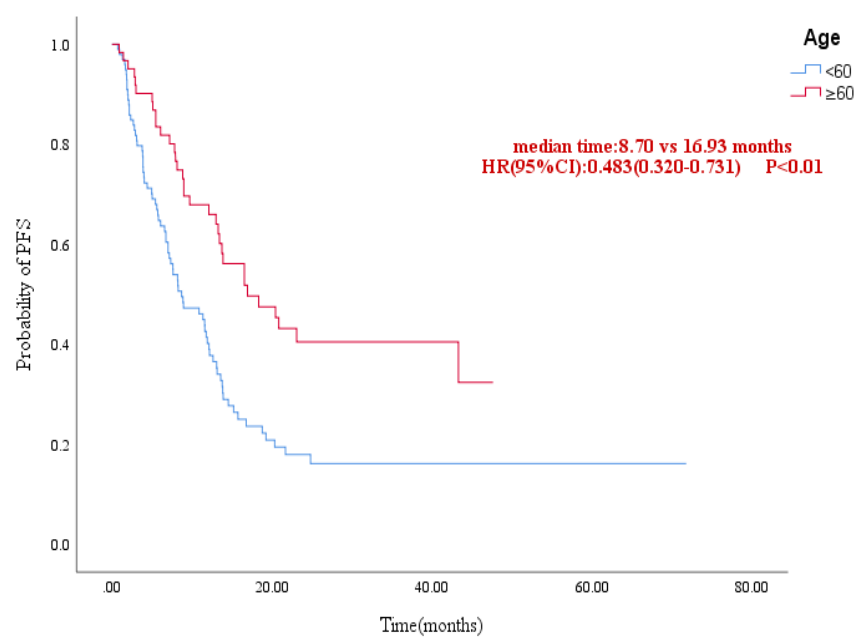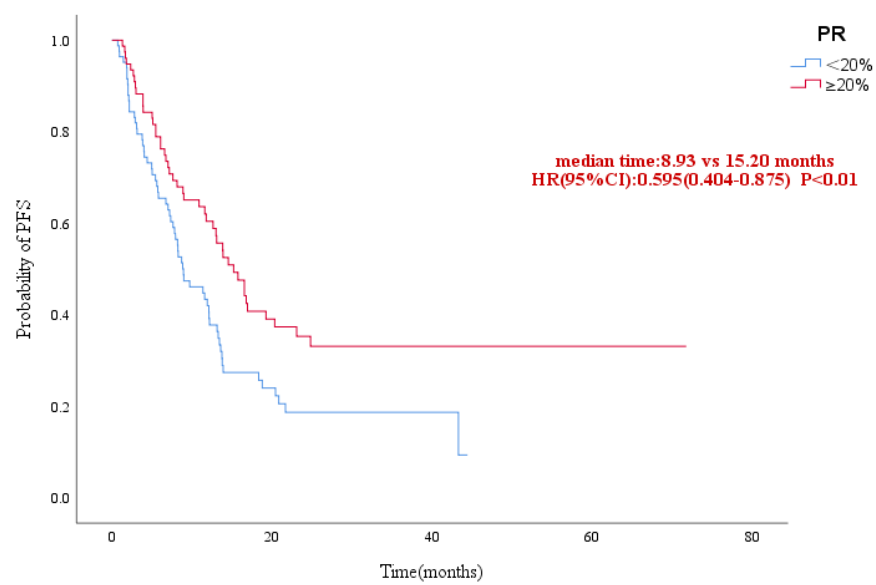

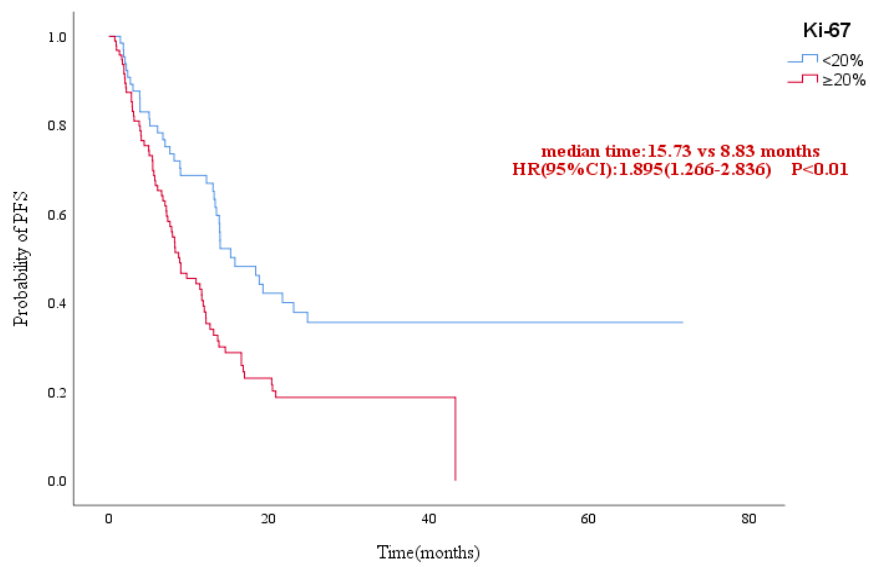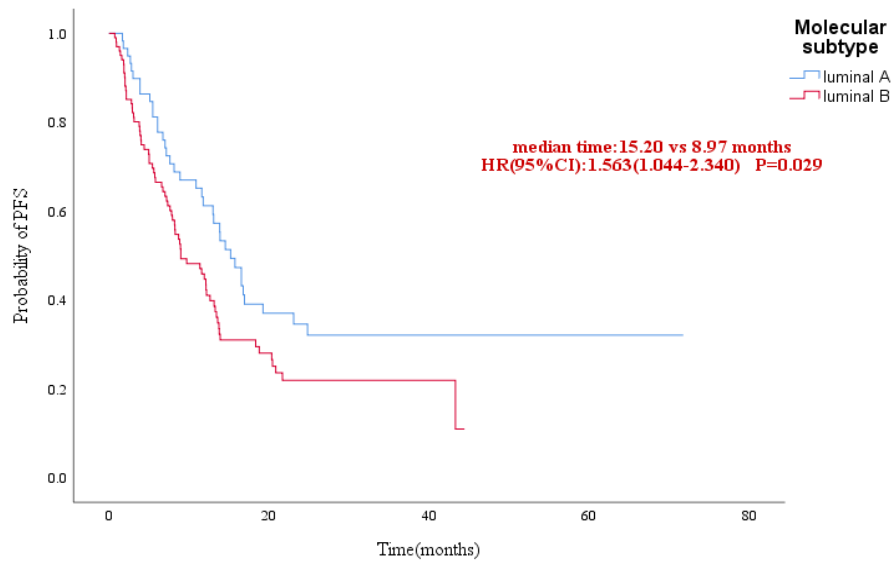

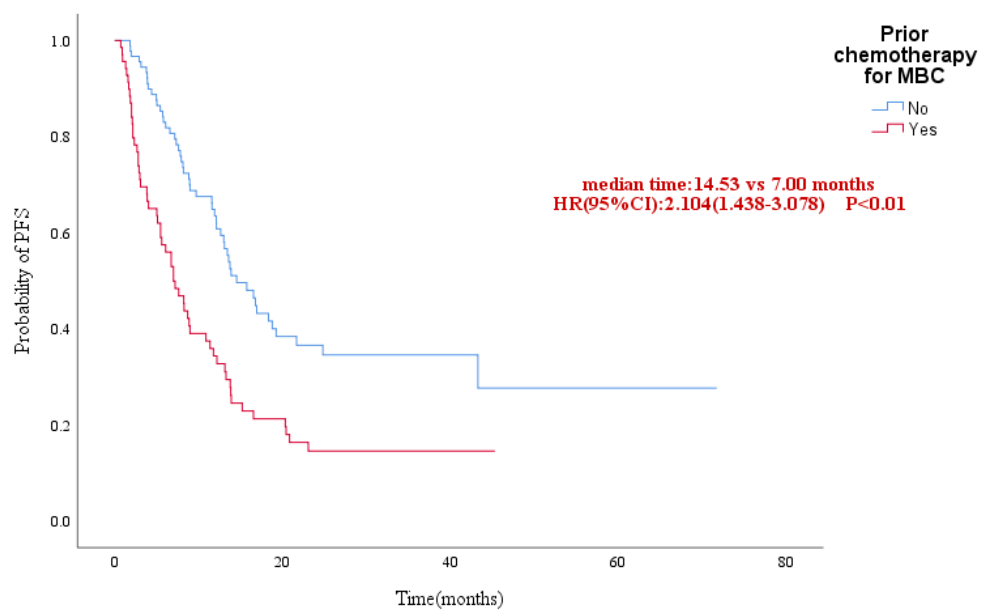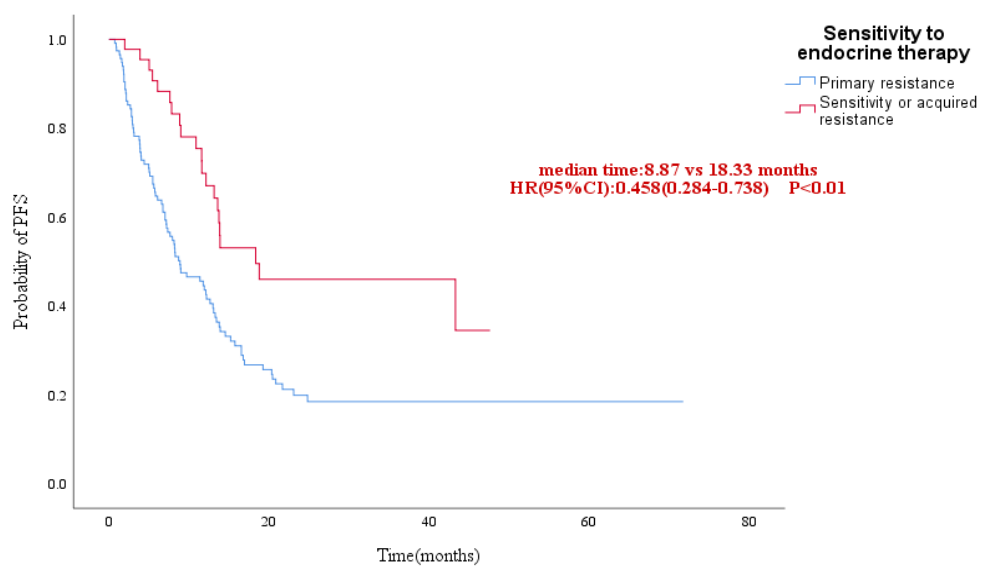

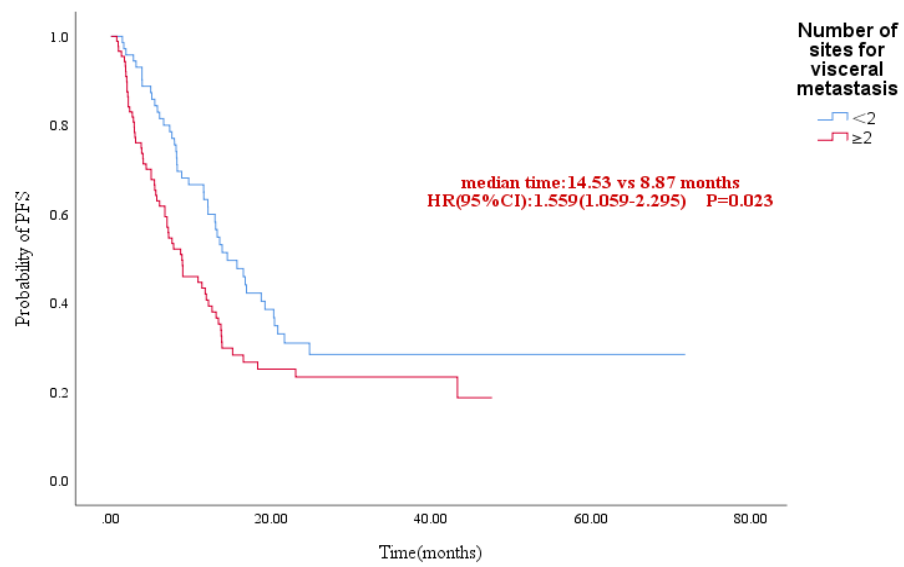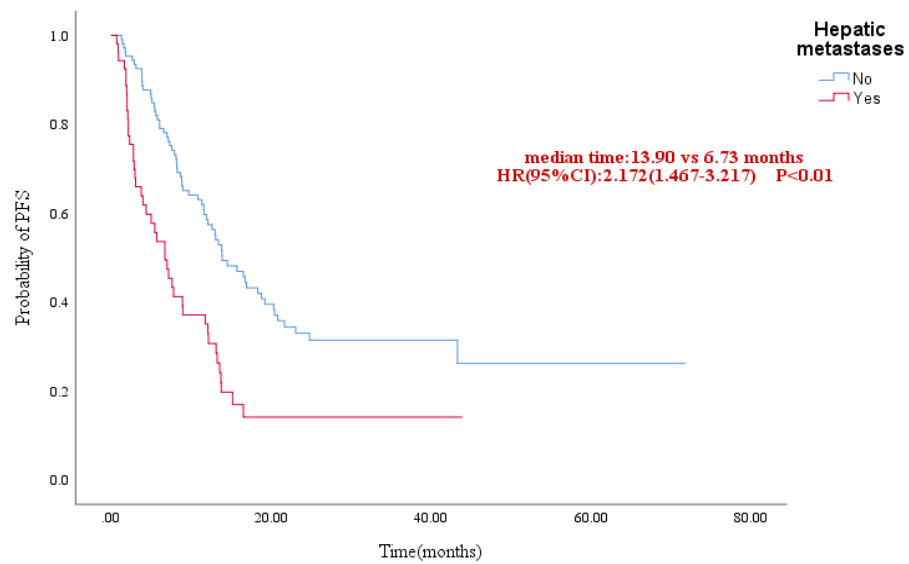

Supplementary Figure 1.PFS comparisons in subgroups

Supplement: Supplementary file 1 — Additional file 1: Supplementary Fig. 1. PFS comparisons in subgroups. [file 12885_2023_11764_MOESM1_ESM.pdf]
